# Functional Outcomes of Cerebellar Malformations

**DOI:** 10.3389/fncel.2019.00441

**Published:** 2019-10-04

**Authors:** Jason S. Gill, Roy V. Sillitoe

**Affiliations:** ^1^Section of Pediatric Neurology and Developmental Neuroscience, Baylor College of Medicine, Houston, TX, United States; ^2^Department of Pathology and Immunology, Baylor College of Medicine, Houston, TX, United States; ^3^Jan and Dan Duncan Neurological Research Institute of Texas Children’s Hospital, Houston, TX, United States; ^4^Department of Neuroscience, Baylor College of Medicine, Houston, TX, United States

**Keywords:** cerebellum, development, Purkinje cell, cerebellar nuclei, circuitry, motor, cognitive

## Abstract

The cerebellum is well-established as a primary center for controlling sensorimotor functions. However, recent experiments have demonstrated additional roles for the cerebellum in higher-order cognitive functions such as language, emotion, reward, social behavior, and working memory. Based on the diversity of behaviors that it can influence, it is therefore not surprising that cerebellar dysfunction is linked to motor diseases such as ataxia, dystonia, tremor, and Parkinson’s disease as well to non-motor disorders including autism spectrum disorders (ASD), schizophrenia, depression, and anxiety. Regardless of the condition, there is a growing consensus that developmental disturbances of the cerebellum may be a central culprit in triggering a number of distinct pathophysiological processes. Here, we consider how cerebellar malformations and neuronal circuit wiring impact brain function and behavior during development. We use the cerebellum as a model to discuss the expanding view that local integrated brain circuits function within the context of distributed global networks to communicate the computations that drive complex behavior. We highlight growing concerns that neurological and neuropsychiatric diseases with severe behavioral outcomes originate from developmental insults to the cerebellum.

## Introduction

Human behavior is seemingly infinite in its functional complexity, yet the human brain is capable of synthesizing sophisticated movements, motivations, emotions, and desires with fluid continuity and into a multitude of distinct, recognizable behaviors. Furthermore, major disruptions affecting large portions of the brain or minor alterations that only subtly set its neural function askew are all immediately evident during disease pathogenesis with the full spectrum of neurological and neuropsychiatric disease readily manifest in altered behavior. How the brain achieves this remarkably robust yet at times fragile integration of sensorimotor and executive function remains a mystery that requires the full intent of neuroscientific inquiry. Despite this monumental task, the synthesis of molecular, cellular, systems, and clinical neuroscience has helped to continuously push our knowledge forward. The cerebellum, now regarded as a central node for the integration of diverse circuit functions, is an interesting starting point to understand how the brain computes and produces the symphonic elegance that characterizes animal behavior.

The cerebellum has traditionally been regarded solely as a regulator of motor function (Manto et al., 2012; Perciavalle et al., 2013; Lang et al., 2017). Over the past several decades, however, a consensus is forming regarding a role for the cerebellum in non-motor behavior, the precise nature of which has yet to be fully elucidated (Koziol et al., 2014; Mariën et al., 2014; Baumann et al., 2015). Turning to clinical observations and literature, a central role for the cerebellum in cognitive function was posited almost 30 years ago (Schmahmann, 1991). In the context of the highly articulated and patterned nature of the cerebellar cortex as well as experimental and clinical observations, the idea of the universal cerebellar transform was conceived (Schmahmann, 2004): motor dysfunction related to cerebellar pathology can manifest as ataxia, dysmetria, dystonia, and tremor, while the cognitive or affective manifestations of these incoordinations may be reflected in pseudobulbar palsy, disinhibition, inattention, and psychosis (Schmahmann, 2004). This mirroring of motor dysfunction in the affective/cognitive sphere is a concept referred to as dysmetria of thought. Thus, regardless of whether the cerebellum is involved in a motor or non-motor behavior, its role in coordinating and integrating different functional modalities is compromised upon insult, and perhaps the compromise of a “universal” neural computation is at fault. This raises at least two intriguing questions: (1) What is the cerebellar circuit architecture that mediates its many functions? (2) Are cerebellar circuits heterogeneous?

Using the cellular composition and anatomy of the cerebellum as a platform, we discuss how developmental, genetic, and mechanical cerebellar disruptions influence circuit assembly and ultimately impact motor and non-motor behavior. To do so, we consider that the cellular composition and cytoarchitecture of the cerebellar cortex, which, while vastly more uniform than the neocortex, is nevertheless anatomically, genetically, and electrophysiologically variable across its microdomains (Cerminara et al., 2015; Apps et al., 2018). The present review will elide the discussion of cerebellar function as a universal cerebellar transform or multiple functionality, instead considering a modified version of the universal cerebellar transform such that as the cerebellum expanded through evolutionary time, it adapted to the increasing requirements associated with behavioral complexity, and concordantly its microarchitecture evolved to have specialized functions that sub-serve distinct cortical areas. At the same time, the cerebellum would have preserved its role in integrating afferent sensory information from the periphery, namely sensory modalities such as vestibular pathways that are evolutionarily well-conserved. There is a possibility that sensorimotor functions are executed by the same circuits that modulate cognitive behaviors (Diedrichsen et al., 2019). In this scenario, the heterogenous anatomy and functional properties of the internal cerebellar microcircuitry could provide such flexibility to occur (Reeber et al., 2013; Beckinghausen and Sillitoe, 2019; Sathyanesan et al., 2019). The present review will seek to synthesize basic neuroscientific and macro-evolutionary observations with human disease observations to help contextualize current efforts in rodent models aimed at developing a more sophisticated understanding of the role of the cerebellum in cognitive and affective behavior. We focus on how these behavioral entities are influenced by first examining how the cerebellum develops its precisely patterned internal architecture and how this cerebellar map drives the assembly and functional architecture of its topographic circuits.

## Cerebellar Cytoarchitecture and Basic Circuitry

In addition to the first descriptions of the Purkinje cell by Johannes Evangelista Purkinje and the potential interactions between cell types suggested by Camillo Golgi using his classic reazione nera or “the black reaction” (described in Herndon, 1963), the earliest extensive descriptions of the complete and precise cellular cytoarchitecture of the cerebellum come from the original studies of Ramon y Cajal, who both revolutionized our understanding of the organization of the nervous system and provided a basis for our current understanding of neuronal transmission (for a review of Cajal’s early findings; see Sotelo, 2008). It is from these pioneering descriptions of Purkinje, Golgi, Cajal and others that the orthodoxy of the uniform cytoarchitecture of the cerebellum arose. It should be noted that Cajal’s findings on the localization and morphology of the different cell types have remained relevant to present day, though with several specialized features of the circuit recently unveiled (e.g., the identification of unipolar brush cells, direct projections from Purkinje cells to granule cells, and the nucleo-cortical projections). In the past 60 years, our understanding of the cytoarchitecture of the cerebellum has thus greatly expanded (Glickstein and Voogd, 1995; Voogd and Glickstein, 1998; Oberdick and Sillitoe, 2011; Ruigrok, 2011; Voogd and Koehler, 2018), with an overarching theme being the demonstration of cellular and circuit heterogeneity.

### A Primer on Cerebellar Circuitry

**FIGURE 1:**
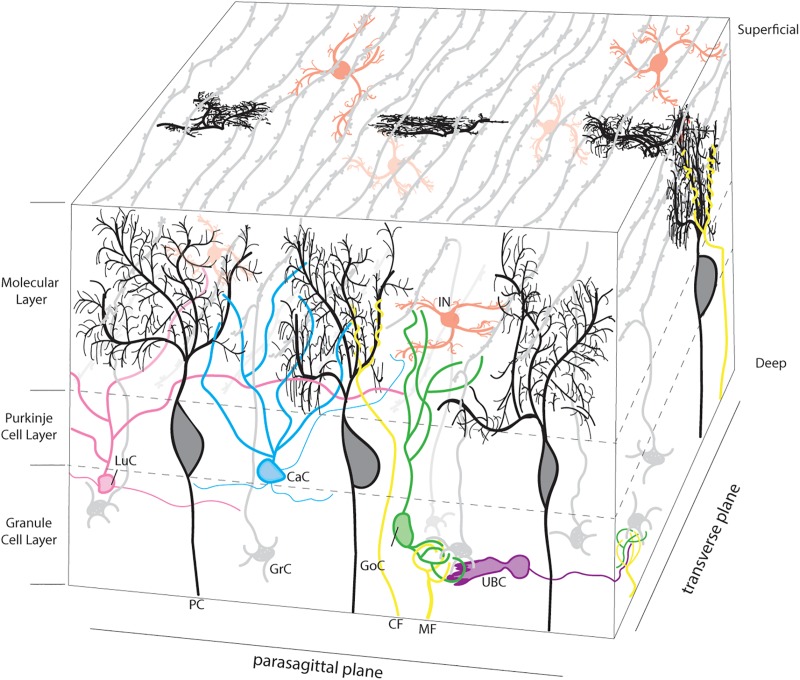
Neuronal microarchitecture of the cerebellar cortex. Representative schema of the layers of the cerebellar cortex. Afferent projections are presented in yellow. Climbing fibers (CF; yellow) project to the molecular layer and target the dendritic tree of a single Purkinje cell (PC; black). Mossy fibers (MF; yellow) terminate in the granule cell layer, forming synaptic connections with granule cells (GrC; gray). The granule cell layer also contains golgi cell (GoC; green), Lugaro cell (LuC; pink), and unipolar brush cell (UBC; purple) interneurons. The cell bodies of granule cells are located in the granule cell layer and project axons to the molecular layer where they branch to form parallel fibers, which run orthogonal to the parasagittal plane. The molecular layer contains the cell bodies of inhibitory interneurons (IIN; red), which include both basket and stellate cells as well as neurites from a variety of neurons, as pictured. Finally, the Purkinje cell layer is occupied by the large cell bodies of the Purkinje cell (PC; black) and the smaller cell bodies of the Candelabrum cells (CaC; Cyan). In 3-dimensional space, the transverse projections of the parallel fibers can integrate and process the afferent information supplied by the climbing and mossy fibers. Basket cell axons, which wrap the Purkinje cell soma and initial segment of the axon, and Bergmann glia palisades that extend into the molecular layer were intentionally excluded to focus on the Purkinje cell anatomy.

**FIGURE 2:**
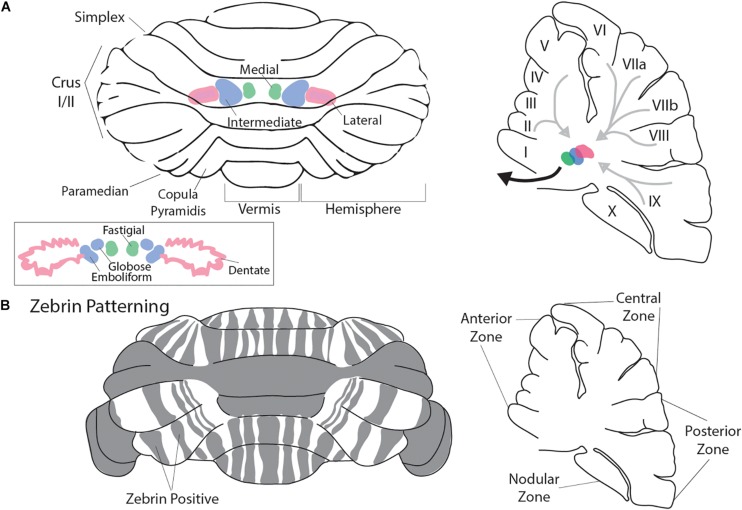
Macroarchitecture of the cerebellum. Rodent cerebellum **(A)** depicted from the dorsal view (left panel). Rostral is superior and caudal is inferior. Vermis is midline. The cerebellar nuclei (CN) are represented in color. In pink is the lateral nucleus, in green is the medial nucleus, and in blue is the intermediate nucleus (**A**; inset). Pictured are the primate cerebellar nuclei: lateral is the dentate (Pink) where the convoluted shape in comparison to the rodent lateral nucleus can be appreciated, medial is the fastigial (green), and the globose and emboliform nuclei (blue; together forming the interposed nuclei) are pictured and are analogous to the murine intermediate nucleus. The right panel presents a parasagittal view of the cerebellum at midline. The cerebellar nuclei are indicated as in the left panel (adapted in part from Sugihara et al., 1999 permission was obtained from Wiley Online Library). Light gray arrows represent schematized Purkinje cell output to the CN, while the heavy black arrow represents CN efferent projections to the cerebral cortex and brainstem. Mouse cerebellum **(B)** depicted from the dorsal view (left panel). Gray indicates representative Zebrin banding pattern. Orientation is as described in **(A)**. The Right panel indicates the mouse cerebellum at midline in the parasagittal plane with zonal nomenclature. Mouse cerebellum adapted from Cerminara et al. (2015). Permission was obtained from Nature research journals.

To appreciate how the cerebellum functions and how it fails in disease, it is useful to recall that connectivity within the cerebellum is understood at a considerable level of detail, with each cell type forming stereotypical connections with its neighbors (Figure 1). The cerebellum has three distinct layers. The most superficial layer, the molecular layer, contains inhibitory interneurons and excitatory climbing fibers. Both project onto the dendritic arbors of Purkinje cells, the cell bodies of which occupy the middle layer called the Purkinje cell layer. The Purkinje cells perform the main computations of the cerebellum. Sandwiched between the Purkinje cells are the very large Bergmann glia. It is important to note that the cerebellar glia are highly heterogenous, although poorly studied (Sotelo and Alvarado-Mallart, 1987; Buffo and Rossi, 2013; Goertzen and Veh, 2018; Wizeman et al., 2019). The Purkinje cell layer also contains interneurons called candelabrum cells. The deepest layer is called the granular layer and it contains billions of excitatory neurons called granule cells as well as the terminals of excitatory mossy fibers that deliver sensory signals to the cerebellar cortex. The granule cell axons, which bifurcate into parallel fibers, are located within the molecular layer. The granular layer also contains inhibitory Golgi cell interneurons, Lugaro cells, and a unique population of excitatory interneurons called unipolar brush cells. The unipolar brush cells are localized mainly in the vermis of lobules IX and X, with a smaller number localized to lobules VI and VII. Although the primary afferent classes are climbing fibers and mossy fibers, there are also modulatory “beaded” fibers that terminate in all layers of the cerebellar cortex and in all ten lobules of the vermis and hemispheres. Below the three layers is a dense network of fiber tracts. Embedded in this network are the cerebellar nuclei (Figure 2A; left panel). The cerebellar nuclei contain specialized neurons that transmit the final output of the cerebellum. Each nucleus is comprised of GABAergic, glycinergic, and glutamatergic cell types. The nuclei are described by their anatomical position in rodents, medial, intermediate and lateral. In primates, the nomenclature is different based on historical anatomic descriptions: the medial nucleus is called fastigial, the intermediate is comprised of the distinct globose and emboliform nuclei which together constitute the interposed nucleus (Figure 2A; inset), and the lateral is called the dentate, which in primates has a complex, convoluted structure (Figure 2A; inset). Together, the cerebellar nuclei link the cerebellar cortex to the rest of the brain and spinal cord (Figure 2A; right panel).

## Cerebellar Heterogeneity: Zones and Topography

The described cerebellar circuitry represents the underlying cellular microstructure that supports the computational domains of the cerebellum, which are comprised of zones (Miterko et al., 2018) that serve to integrate sensorimotor information into executive output, both motor and cognitive. In the second half of the twentieth century, great advances were made in elucidating the circuits of the cerebellum, culminating in the conceptualization of cerebellar modules which were initially based on precise characterizations of the neuroanatomy of the cerebellum (Voogd, 2011). During this time, characterization of afferent olivocerebellar projections to the cerebellar hemispheres as well as efferent dentatothalamocortical projections revealed a parasagittal zonal architecture (van Rossum, 1969; Courville et al., 1974; Matsushita and Wang, 1987). These anatomical findings accompanied the first molecular characterization of the parasagittal zonal configuration of Purkinje cells using 5′ nucleosidase (Scott, 1963). This would be a herald to the molecular delineation and characterization of the cerebellum, the knowledge of which has been vastly expanded with function and molecular genetics over the last 40 years (Apps and Hawkes, 2009).

The most well characterized molecular marker of cerebellar zones is Zebrin II (Hawkes and Leclerc, 1987; Brochu et al., 1990; Ahn et al., 1994). Zebrin II is expressed by a subset of Purkinje cells in highly conspicuous and evolutionarily conserved parasagittal bands (Figure 2B; left panel; Sillitoe et al., 2004; Apps and Hawkes, 2009). Initial characterizations of Zebrin II refined the anatomical descriptions of the zonal architecture of the cerebellum (Voogd et al., 2003), revealing a complex parasagittal organization with diverging and coalescing molecularly defined longitudinal zones. Further supporting a notion that the parasagittal zonal organization is a functional component of the cerebellum, numerous additional molecules expressed in subsets of Purkinje cells have been found to colocalize with Zebrin II (e.g., EAAT4, PLCβ3; Dehnes et al., 1998; Sarna et al., 2006), or to have a complementary staining pattern to Zebrin II (e.g., mGluR1β, PLCβ4; Mateos et al., 2001; Sarna et al., 2006). While less thoroughly characterized, there is also patterned expression of molecules expressed in cerebellar interneurons, namely the unipolar brush cells, granule cells, and Golgi cells (Consalez and Hawkes, 2013).

As described above, the initial observations of zonal architecture of the cerebellum were anatomic rather than molecular, yet subsequent analyses would buttress the initial anatomic observations with molecular data that helps to elucidate the functional role of the zonal architecture. In addition, numerous studies have contributed to an understanding of zonal architecture whereby there is a segregation of afferent projections, including both mossy fibers and climbing fibers, that have a complex correspondence to the parasagittal organization of Purkinje cell zones (Sugihara, 2004; Pijpers et al., 2006; Sawada et al., 2008; Armstrong et al., 2009; Paukert et al., 2010; Gebre et al., 2012). The parasagittal zonal architecture of the cerebellum doesn’t independently comment on the nature of the computations performed by the cerebellar modules. In fact, modules integrating diverse afferent information may be elegant loci in which integrative computations are performed, such as those called for in embodied cognition (Guell et al., 2018). Consistent with the anatomic observations in which there are entire populations of cell types that have restricted localization along the anterior-posterior axis in the cerebellum (Braak and Braak, 1993; Mugnaini and Floris, 1994), there are also Purkinje cell specific molecular markers that have restricted expression along the anterior-posterior axis, namely D3 dopamine receptor, dopamine transporter (DAT), and synaptic vesicular monoamine transporter (VMAT) (Kim Y.S. et al., 2009). Further investigations into the functional relevance of these indices of cerebellar heterogeneity may offer insight into how the cerebellum integrates or processes the wide range of afferent information that it receives.

As might be expected, given the anatomic and molecular heterogeneity found across the cerebellum, the neurophysiology of the cerebellar cortex is not uniform. Corresponding to the variation in D3, DAT, and VMAT along the anterior-posterior axis (Kim Y.S. et al., 2009), there are high levels of depolarization induced slow currents (DISCs) in the Purkinje cells of the posterior lobe vermis while they are seen only at low levels in the Purkinje cells of the anterior vermis (Shin et al., 2008). In addition, Purkinje cells in lobules III/IV have differences in passive and active membrane properties as compared to those in lobule X, resulting in lobule X Purkinje cells being less excitable and displaying a greater variety of firing patterns in response to depolarizing currents (Kim et al., 2012). Finally, even functional connectivity has been shown to vary based on anatomic location, with the finding that there is direct inhibition of granule cells by Purkinje cells in a lobule dependent manner (Guo et al., 2016).

In the mediolateral axis, it has been shown that activation of mGluR and the associated synaptic plasticity in response to complex spikes is reduced in Zebrin II + Purkinje cells (Wadiche and Jahr, 2005), and is most likely mediated by EAAT4 expression and function (Brasnjo and Otis, 2001). Furthermore, the firing properties of Purkinje cells vary based on whether they are located in Zebrin II + or Zebrin II – zones (Xiao et al., 2014; Zhou et al., 2014), although these distinctions may occur in a region specific manner rather than being a general feature of Zebrin II ± zones throughout the cerebellar cortex. Moreover, it remains unclear how zones relate to behavior (Horn et al., 2010; Cerminara and Apps, 2011), particularly whether each zone has a dedicated behavior (or set of behaviors) and if a given zone can contribute to both motor and non-motor behavior. Regardless, it seems plausible that a segmentation of the cerebellum into a map could provide the flexibility through which different behaviors are executed. Having provided an overview of the micro and macro architectural features of the cerebellum, a discussion of the developmental processes that underlie the elegant structure of the cerebellum follows.

## Genes, Molecules, and Morphogenesis During Cerebellar Development

There are several morphogenetic stages that form the cerebellum. The neural tube divides into morphological divisions called neuromeres, including the forebrain, midbrain, and hindbrain. In the hindbrain, the coordinated action of various transcription factors and the mobilization of secreted morphogens demarcate the neuroepithelial territory that will give rise to the cerebellum and specify its neurons and glia. The mouse cerebellar primordium arises between E8.5 and E9.5 from within the metencephalon (Wassef and Joyner, 1997; Zervas et al., 2004). Initially, abutting expression domains of the mutually repressive homeobox genes *Orthodenticle homolog 2* (*Otx2*) and *Gastrulation brain homeobox 2* (*Gbx2*) regionalize the mid-hindbrain boundary (MHB) and form the isthmic organizer (IsO). The IsO secretes Fibroblast growth factor 8 (Fgf8), which is necessary and sufficient for the differentiation of cerebellar cells and the initiation of its gross morphology (reviewed in Zervas et al., 2005). *Fgf8* controls cerebellar development through its inductive power and its ability to activate and cooperate with genes such as *engrailed 1* (*En1*), *engrailed 2* (*En2*), as well as the paired box genes *Pax2* and *Pax5* (reviewed in Sillitoe and Joyner, 2007). Once the cerebellar territory is demarcated, cell lineages are committed in the germinal zones. There are two germinal zones that produce the different cerebellar cell types: the rhombic lip and the ventricular zone. The rhombic lip is located at the dorsal and extreme posterior aspect of the cerebellum. Genetic fate mapping using *Atoh1* alleles in mouse showed that the rhombic lip gives rise to all cerebellar glutamatergic neurons including the large projection neurons of the cerebellar nuclei, granule cells, and unipolar brush cells (Wingate, 2001; MacHold and Fishell, 2005; Wang et al., 2005; Englund et al., 2006). The other germinal zone is called the ventricular zone; it lines the base of the fourth ventricle. The ventricular zone generates all the GABAergic neurons of the cerebellum including the different classes of interneurons, the inhibitory cerebellar nuclei neurons, and all the Purkinje cells. The GABAergic neurons are specified from progenitors that express the transcription factor-encoding gene *Ptf1a* (Hoshino et al., 2005; Pascual et al., 2007). However, unique cell identities derive from the patterning of both germinal zones into multiple molecular domains in the rhombic lip (Machold et al., 2007; Chizhikov et al., 2010; Green and Wingate, 2014; Yeung et al., 2014) and the ventricular zone (Chizhikov et al., 2006; Zordan et al., 2008; Lundell et al., 2009; Seto et al., 2014). The mechanism by which the different pools of neuronal progenitors give rise to the distinct cell types of the cerebellum are not fully understood. However, using Purkinje cells as an example, it is estimated that the entire Purkinje cell population in the adult arises from ∼100 to 150 precursors, and their specification occurs at around E7–E8 in mice (Baader and Schilling, 1996; Mathis et al., 1997; Hawkes et al., 1998; Watson et al., 2005). Although it is not clear if Purkinje cell precursors are restricted to different sub-lineages, there is evidence that after differentiation, Purkinje cells become restricted to distinct subsets that fall into the pattern of stripes and zones (Figure 2B; Hawkes and Gravel, 1991; Hawkes and Eisenman, 1997; Oberdick et al., 1998; Armstrong and Hawkes, 2000; Herrup and Kuemerle, 2002; Larouche and Hawkes, 2006; Sillitoe and Joyner, 2007; White and Sillitoe, 2013). The Purkinje cell patterns may guide the development of cerebellar motor and non-motor circuits (Sathyanesan et al., 2019), and disrupting cerebellar patterning could lead to a wide variety of conditions (Reeber et al., 2013). Motor conditions with cerebellar involvement include ataxia, dystonia, and tremor, and non-motor disorders include schizophrenia, Tourette’s, and autism spectrum disorders (ASD).

The above discussion on micro- and macroarchitectural development and patterning of the cerebellum through development was almost exclusively carried out in model organisms due to their genetic and experimental tractability. As will be discussed later in the section entitled “An evolutionary perspective of gross cerebellar architecture”, the cellular architecture of the cerebellum has been largely conserved across vertebrate evolution. However, there have been important gross adaptations in the cerebellar architecture of primates as compared to the murine cerebellum. Interestingly, the massive expansion of the primate cerebellum has been largely related to widespread increases in surface area due to increased foliation as well as the more focal lateral expansion of the cerebellar hemispheres (Balsters et al., 2010). This has occurred in tandem with increased surface area of the primate dentate nucleus [Figure 2A, inset (Dentate), compare to Figure 2A (Lateral); (Sultan et al., 2010)] as well as the expansion of the primate neocortex (Balsters et al., 2010). The functional implications of these morphologic differences may be alluded to in human cerebellar diseases. As a result, we will now turn to ASD as a model condition to discuss how cerebellar development, particularly at the nexus of genetics, morphogenesis, and circuit wiring, impacts functional outcomes. Subsequently, we highlight the extensive inter-regional connectivity of the cerebellum and how it may explain the pervasive effects of cerebellar disruptions.

## The Cerebellum in Human Affective and Cognitive Diseases

ASD encompass a broad multi-etiologic domain that converges on a characteristic disruption of normal social behavior with a relative sparing of motor function. The term “autism” was coined in the early parts of the twentieth century by Eugen Bleuler to describe a seeming withdrawal from the outside world into the “self” by schizophrenic patients (Lai et al., 2014). The term was then adapted by Leo Kanner to describe the pediatric developmental condition that today is recognized as ASD, drawing a parallel between the withdrawal into the self-seen in schizophrenia to the inability of ASD patients to relate to others (Kanner, 1943). Since then, a more holistic understanding of autism has come to bear. In terms of symptomatology, this has coincided with the recognition that ASDs encompass a wide variety of phenotypes, which may include significant affective, cognitive, and motor impairments (Lai et al., 2014). Furthermore, there is an equally wide spectrum of etiologies, ranging from single gene disruptions with mendelian inheritance, to heritable non-mendelian disease, to entirely acquired etiologies (Lai et al., 2014). However, even in the cases of ASD with mendelian inheritance, there is a varying degree of penetrance, emphasizing the complex genetic and environmental contributions to the disease presentation. Equally complex are the regions of the brain that could drive the behavioral defects. Here, we will examine the ASDs from the perspective of the cerebellum, which has been implicated extensively in ASD (Fatemi et al., 2012), with recent experimental data further supporting its contribution.

**FIGURE 3:**
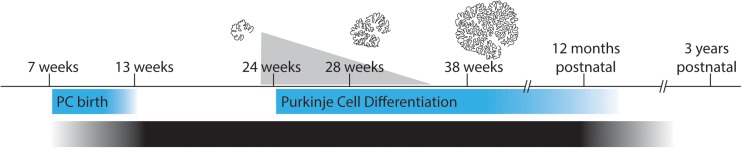
Purkinje cell differentiation, cerebellar expansion, and sensitive periods for developmental disorders. Pictured are various correlations along the time line of gestation through early childhood. Pictured in the upper portion of the figure is a scaled representation of cerebellar size and foliation during gestation. Adapted from Rakic and Sidman (1970) with permission obtained from Wiley Online Library. The superimposed gray triangle represents the incidence of developmental comorbidities associated with premature birth at the corresponding gestational ages. Below the time line in blue are the gestational ages of Purkinje cell birth and differentiation. (Bottom) A gradient showing the time period during which all etiologies of autism are thought to occur. Figure adapted in part from Sathyanesan et al. (2019) with permission obtained from Nature research journals.

While many brain regions have been found to be disrupted in patients with ASD, one of the most consistent sites of brain pathology in ASD is the cerebellum (Allen, 2005). Furthermore, reduction in the normal number of Purkinje cells was one of the most frequently reported abnormalities in early studies (Bailey et al., 1998; Kemper and Bauman, 1998). However, it has been difficult to determine at which point in development Purkinje cell loss occurs, or how much cell loss must occur in order to contribute to the development of ASD symptoms. It is important to mention at this time the developmental correlation between the cerebellum and ASD such that both the environmental insults and brain injuries that increase risk for ASD indeed occur at the time at which there is a massive expansion of the cerebellum (Wang et al., 2014; Figure 3), namely the third trimester of pregnancy continuing into the early postnatal period. Furthermore, cerebellar injury during this sensitive period confers a significant predisposition to neurocognitive disability, including the development of ASD (Limperopoulos et al., 2007). In fact, the risk conferred by cerebellar injury at birth to development of ASD was up to 36x (Wang et al., 2014).

How do these developmental features relate to the Purkinje cell pathology noted in post-mortem studies of autistic individuals? Several lines of evidence provide circumstantial evidence for the timing of Purkinje cell dysfunction/death. First, it has been reported that cerebellar stellate and basket cells are preserved in number (Whitney et al., 2009), which is an indication that Purkinje cells likely migrated and settled appropriately after their genesis. Second, in post-mortem studies there is no alteration in the number of inferior olivary neurons, which would be expected to undergo degeneration in the context of denervation from cell death (see related discussion on hypertrophic olivary degeneration; Fatemi et al., 2012), indicating that the Purkinje cell loss occurs prior to the innervation of the Purkinje cells by climbing fiber afferents from the inferior olive in the immediate perinatal period [although, the mode of insult and speed of Purkinje cell loss could affect how their target climbing fibers respond (Rossi and Strata, 1995)]. More recent analysis, however, has called into question how generalized the phenomenon of Purkinje cell loss is in autistic patients, finding that some autistic patients fail to show alterations in Purkinje cell number (Whitney et al., 2008). Given the very wide etiologic spectrum of ASD, perhaps it is not surprising to find that there are manifestations of ASD that do not involve Purkinje cell loss. Nonetheless, available evidence suggests that at least some forms of ASD are associated with loss of Purkinje cells at a critical period after proliferation and migration and prior to completion of circuit wiring of the cerebellum into its mature architecture with functioning connections. It should also be noted though that compensation, both at the genetic and cellular level, could play a role in determining the final number of cells in affected patients.

One of the most striking aspects of ASD is the abnormal social and emotional behavior of affected individuals; a major source of distress in the disease presentation to the parents of an autistic child is the absence of affection. Is there, then, an association between the cerebellum and affect? Recently, in fact, there has been increasing awareness of a role for the cerebellum in affective behavior (Ackermann et al., 1998; Steinlin, 2007). Further, over 40 years ago, while investigating therapies for medically refractory neuropsychiatric illness, it was found that stimulation of the vermis could ameliorate aggressive behavior in patients (Heath, 1977). These findings were functionally extended, showing that modulation of the vermis and the associated fastigial nuclei altered neuronal activity in the deep limbic circuit including in the hippocampus, amygdala, and septal region (Heath et al., 1978). In rats, mechanical disruption of the vermis in pups was found to produce disruption of social and emotional behavior in adulthood (Bobee et al., 2000). Numerous imaging studies in humans have found associations between affect and vermis activation (Tavano et al., 2007; Stoodley and Schmahmann, 2010; Buckner et al., 2011). Specifically, positron emission tomography (PET) and functional magnetic resonance imaging (MRI) studies have found that activation of the vermis is correlated with induced anxiety (Reiman et al., 1989), grief (Gündel et al., 1946), unpleasant emotions (Lane et al., 1997), and depression (Beauregard et al., 1998). Finally, acquired lesions of the vermis in adults have been found to lead to affective disturbances, including emotional lability or pseudobulbar palsy in a patient with a midline cerebellar cyst (Parvizi and Schiffer, 2014) and dysphoria and affective flattening in a patient with isolated cerebellar ischemic stroke (Paulus et al., 2004).

The frank disruption of normal affective behavior is one of the hallmarks of ASD, which heavily implicates vermis involvement as a pathogenic mechanism in cerebellar etiologies of ASD. In fact, a wide range of early studies, looking at post-mortem tissue and using imaging modalities, found disruptions of the vermis in affected individuals. What did these studies reveal? There are findings of hypoplasia of vermal lobules VI and VII (Courchesne et al., 1988), decreased Purkinje cell density in the vermis (Ritvo et al., 1986), mixed hypoplasia and hyperplasia in affected individuals (Courchesne et al., 1994a, b), and overall diminished volume of the vermis (Courchesne et al., 2001). A more recent analysis using volumetric processing of MRI in affected individuals was more equivocal, but did find that overall vermis volume was significantly decreased in ASD patients (Scott et al., 2009).

When looking at gross changes in brain structure, there are a wide array of findings that will not be discussed in great detail, though several key points should be made. First, it is important to note that changes in brain volume are often not stable and can vary significantly over time, based on anatomic location, and based on sex (see discussions in Stanfield et al., 2008; Sussman et al., 2015). In particular, changes in volume have been seen in the neocortex, limbic structures, and the corpus callosum (Stanfield et al., 2008). Additionally, there can be a tendency for early expansion of gray and white matter, with later diminishing of volume (Courchesne et al., 2001). As a result, time of study, sex of participant, and IQ of participant are all independent variables that may affect brain volume analysis without consideration of etiology of ASD in that patient. Regarding the cerebellum, volumetric changes have been reported in numerous studies over the last four decades, but it remains unclear how cerebellar white matter versus cerebellar gray matter are affected in ASD, or whether one is a better indicator of pathophysiology over the other.

Nonetheless, the evaluated studies heavily implicate the cerebellum and cerebellar pathology in ASD, however, they do not comment to a great degree on the functional implications of this pathology. For a better understanding from this perspective, a deeper look at structural and functional network integrity in individuals with ASD vis a vis the cerebellum will be helpful.

While the above section focused on the affective changes that characterize ASD, it is important to note that ASD is a pervasive developmental disorder that may have subtle or gross deficits in all domains (Lauritsen, 2013). In correspondence with these observations, aberrant structural and functional connectivity has been observed throughout the cerebellum as well as in the cerebellar efferent pathways that project to the cerebral cortex (D’Mello and Stoodley, 2015). In particular, when controlled for age and IQ, children with ASD showed alterations in cerebellar white matter integrity (Sahyoun et al., 2010). Similar alterations have been in found in the inferior, middle, and superior cerebellar peduncles (SCP), which are the massive white matter afferent and efferent tracts that link the cerebellum with the rest of the nervous system (Catani et al., 2008; Sivaswamy et al., 2010). A caveat to these and the following discussed studies is that the comparisons made were often between individuals with high functioning autism and typically developing individuals due to the required use of imaging modalities in awake, cooperative patients. As a result, whether this level of analysis underestimates the connectivity changes that might be seen in more severe autism, reflects a subset of patients with a fundamentally different etiology of ASD, or is broadly generalizable to all individuals with ASD is unclear.

The imaging studies discussed above used diffusion tensor imaging (DTI) to evaluate white matter structural integrity. Further analysis of cerebellar function in autistic individuals has been conducted using assays of functional connectivity (FC), which uses correlated activity between brain regions in awake patients undergoing functional magnetic resonance imaging (fMRI). Consistent with the structural findings described above, there is abnormal functional connectivity between the cerebellum and the cortex in ASD individuals (Noonan et al., 2009; Khan et al., 2015). However, unexpectedly, both increased and decreased connectivity can be seen, depending on the brain region evaluated. In particular, it was reported there is an increased connectivity between the anterior cerebellum and the sensorimotor areas of the cortex, while there is a decrease in FC between the cerebellar hemispheres and the supramodal, or cognitive, areas of the cortex (Khan et al., 2015). A second study found that the patterns of connectivity in ASD compared to typically developing children were similar, but more extensive in the ASD population (Noonan et al., 2009). Regarding cerebellar gray matter, reductions have been found in both the posterior vermis as well as the cerebellar hemispheres (Stoodley, 2014; Mello et al., 2015).

Linking the findings of reduced cerebellar gray matter, the elaboration of the cerebellum in the perinatal period, and aberrant functional connectivity in ASD, recent transcriptomic profiling of Purkinje cells during development found that there is an enrichment of genes associated with ASD (Clifford et al., 2019). Interestingly, when compared to ASD-associated genes expressed during neocortical development, those in the Purkinje cell cluster were less likely to be associated with comorbid intellectual disability (Clifford et al., 2019). How this finding fits with other analyses linking high functioning, but not low functioning, ASD with cerebellar pathology warrants additional investigation (Noonan et al., 2009; Scott et al., 2009; Khan et al., 2015). In context of the imaging studies described above, as well as earlier studies examining post-mortem tissue, perhaps the variability in cerebellar pathology reflects the wide range of etiologies that contribute to ASD. Nonetheless, the growing body of literature associating the cerebellum with ASD supports the role of normal cerebellar function in all forms of behavior, motor and non-motor alike.

As mentioned previously, cerebellar injury during the period of cerebellar expansion (3rd trimester to birth) confers a significant risk (up to 36x compared to the general population) for neurocognitive dysfunction and ASD (Limperopoulos et al., 2007; Wang et al., 2014). A recent case report highlighted this association, whereby a child with history of bilateral intrauterine cerebellar stroke underwent neurocognitive evaluation and was diagnosed with ASD (Whiting et al., 2019). Further analysis of patients with congenital cerebellar malformations has revealed consistent non-motor deficits, with these studies demonstrating some degree of anatomic predictability, with vermal malformation showing a predilection for affective dysregulation and hemispheric malformations showing a predilection for executive and linguistic deficits (Tavano et al., 2007). However, in addition to the examination of purely congenital malformations, acquired lesions of the cerebellum, namely resection of low grade posterior fossa tumors, has also been associated with cognitive impairments (Levisohn et al., 2000; Beebe et al., 2005).

The most extensively studied clinical entity related to invasive damage of the cerebellum is posterior fossa syndrome (PFS) related to resection of medulloblastoma. The posterior fossa is the most common location of pediatric brain tumors, and among those tumors in particular, and all pediatric brain tumors generally, medulloblastoma is the most frequent (Pollack, 1994; Northcott et al., 2012). A key component of medulloblastoma management is surgical gross total resection, and the extent of the resection is both a prognostic factor for progression-free survival, but also the development of PFS (Zeltzer et al., 1999; Korah et al., 2010). PFS is a perisurgical constellation of symptoms characterized primarily by mutism, with a wide range of variable accompanying signs including emotional lability, ataxia, hypotonia, and behavioral disturbances (Rekate et al., 1985; Gudrunardottir et al., 2011; Lanier and Abrams, 2017). The overt symptoms of mutism, ataxia, and hypotonia often resolve spontaneously in the months following surgery (Gudrunardottir et al., 2011). However, there has recently been an increasing understanding that the occurrence of PFS is an independent predictor of significant long-term neurocognitive dysfunction in a wide variety of domains, including general intellectual ability, processing speed, attention, working memory, and spatial relations – more so than radiation exposure (Palmer et al., 2010; Schreiber et al., 2017). The majority of the literature fails to reach a consensus on the precise etiology of PFS and there are a wide range of theories including both cerebellar and non-cerebellar causes (Lanier and Abrams, 2017). However, as mentioned previously, the non-motor sequelae of resection of low grade tumors from the cerebellar parenchyma implicates the cerebellum in the etiology of PFS, as does the association of PFS with ataxia (Levisohn et al., 2000; Beebe et al., 2005).

Numerous studies have been undertaken to evaluate the changes in cerebellar structure and connectivity in patients who have experienced PFS (Morris et al., 2009; Patay et al., 2014; Avula et al., 2016; Toescu et al., 2018a, b). An evaluation of factors predisposing medulloblastoma patients to development of PFS found that tumor location proximal to the SCP made development of PFS more likely (Morris et al., 2009). This finding was consistent with the subsequent finding that immediate post-operative imaging revealing evidence of disruption of the proximal dentatothalamocortical efferent tracts was a consistent feature in patients with PFS (Morris et al., 2009). Furthermore, compared to patients who did not develop PFS, those who experienced PFS had a consistent alteration in white matter integrity based on DTI of the bilateral SCP, as well as white matter changes in associated non-sensorimotor cortical areas (Morris et al., 2009). The involvement of the proximal dentatothalamocortical pathway due to damage to the SCP was replicated in a subsequent study using conventional MRI image processing [namely fluid-attenuated inversion recovery (FLAIR) and diffusion weighted imaging (DWI]) (Toescu et al., 2018b). This study also found changes in the dentate nucleus in patients with PFS, though this appeared to be a late onset pathology, rather than an acute problem tightly associated with behavioral morbidity (Toescu et al., 2018b). Consistent with PFS being associated with widespread disruption of cerebellar function, multiple studies have found a significant association of PFS with subsequent bilateral hypertrophic degeneration of the olivary nuclei (Patay et al., 2014; Avula et al., 2016). The inferior olive is involved in conveying afferent information to the cerebellum, via climbing fibers, as well as providing feedforward modulation of cerebellar output carried by the dentatorubral afferents. This dentatorubro-olivary loop is referred to as the triangle of Guillain and Molleret. While the debate on the mechanisms involved in the development of PFS are not resolved, there is a significant and growing body of literature implicating disruption of the cerebellar efferent pathways (Toescu et al., 2018a).

Together, the reviewed PFS data offer two salient observations regarding the role of the cerebellum in non-motor function. First, immediate mechanical damage to the cerebellar efferent pathways leads to a profound disruption of cerebellar function manifesting as ataxia, mutism, and emotional lability, among other clinical findings that comprise clinical PFS. Second, the initial disruption and alteration of cerebellar pathways lead to a second more permanent disruption of function which persists for many years as neurocognitive dysfunction and is reflected in dentato-olivary gray matter degeneration and widespread alterations in cortical white matter structures. This second more permanent alteration, interestingly, manifests almost entirely as neurocognitive rather than motor deficits. Subsequent work aimed at understanding why certain clinical signs and symptoms of PFS resolve while neurocognitive changes persist will be invaluable. Furthermore, a detailed look at how the integrity of the dentato-thalamo-cortical and dentato-rubro-olivary pathways contribute to maintaining the integrity of the inferior olive and dentate nucleus, as well as cortical white matter, may be a valuable contribution to the understanding of cerebellar function.

In concurrence with recent consensus discussions (Koziol et al., 2014; Baumann et al., 2015), there is a prominent and long-standing implication of the cerebellum in non-motor function. However, a great deal of the reviewed body of literature are based mainly on correlative analyses. Lacking until recently has been a direct functional understanding of the cerebellum in non-motor domains from the perspective of neurophysiology, cerebellar computations, and anatomic connectivity (Wagner et al., 2017; Watson et al., 2018; Carta et al., 2019; McAfee et al., 2019). Here, we will discuss a few select studies that have used rodent models and various manipulations to understand the nexus of basic and clinical neuroscience of the cerebellum as it relates to cognition functions.

## Mouse Models of Cerebellar Cognitive Disorders

One of the early genes to be associated with ASD in humans was the homeobox-encoding gene ENGRAILED (EN) (Petit et al., 1995). Even at that time, there was a noted association between ASD and the cerebellum, but the precise nature of how EN was involved in ASD was unclear. Clinically, genome wide association studies continued to solidify the correlation between EN and ASD (Benayed et al., 2005, 2009). To better understand the pathophysiology of EN mutations in ASD, it would be instructive to discuss a series of studies that examined the role of EN during cerebellar patterning. It was initially observed that disruption of EN signaling in mice led to subtle alterations in the gross morphology of cerebellar lobules across the AP axis (Joyner et al., 1991). Subsequent analysis found that more pronounced molecular disruptions of the parasagittal zonal patterning also could be seen in various EN mutant lines (Sillitoe et al., 2008). These studies clearly defined a role for EN signaling along two major cerebellar axes, which could be independent, thus positing that AP and medio-lateral (ML) patterning were independently coordinated. Subsequently, it was found that EN signaling was also required for proper targeting of cerebellar mossy fiber afferents (Sillitoe et al., 2010). Building on the original EN association studies (Gharani et al., 2004), a recent report has confirmed the importance of EN signaling in the manifestations of ASD, while also finding that the clinical manifestation of the disease was not tightly associated with the particular polymorphism expressed in a given affected patient (Carratala-Marco et al., 2018). This may not be surprising given the complexity of somatotopy in cerebellar function (Apps and Hawkes, 2009) as well as the finding that multiple molecular mechanisms, not just EN, are required for cerebellar patterning in both the AP and ML axes (Sillitoe and Joyner, 2007; White and Sillitoe, 2013). Further correlations of EN associated disruptions in the mouse cerebellum with particular behavioral outcomes and neurophysiological alterations of the cortex may be informative in fully understanding how the cerebellum mediates non-motor behavior. In this regard, it is interesting that EN is associated with hippocampal and cortical function (Sgadò et al., 2013), and cerebellum-hippocampal and cerebellar-cortical interactions mediate a number of non-motor behaviors (Watson et al., 2018; McAfee et al., 2019).

While the studies of EN commented on mechanisms involved in disrupting the patterning and development of the cerebellum, more recent studies aimed at analyzing how aberrant Purkinje cell function may lead to ASD utilized targeted mutational analysis by producing Purkinje cell specific tuberous sclerosis complex (TSC) mouse models. TSC is an autosomal dominant disorder comprised of mutations in two genes, TSC1 or TSC2, which results in aberrant signaling in the mechanistic target of rapamycin (mTOR) pathway. mTOR is a key pathway involved in protein synthesis, cell proliferation, and cell growth (Sundberg and Sahin, 2015). In addition to epilepsy and complications to tuber formation throughout the body, approximately 50% of TSC patients meet the criteria for diagnosis of ASD, with a substantial portion of others suffering more subtle neuropsychiatric disturbances (Sundberg and Sahin, 2015; Gipson and Johnston, 2017). Though a previous study linked cerebellar tuber formation to the development of ASD (Weber et al., 2000), more recent efforts have been made to investigate how Purkinje cell function is specifically disrupted through targeted knock-out of TSC1 in Purkinje cells (Tsai et al., 2012; Stoodley et al., 2018). Initial analysis showed that when TSC1 is knocked out of Purkinje cells, there are substantial morphological and neurophysiological changes in Purkinje cells (Tsai et al., 2012). These changes were sufficient to cause behavioral anomalies consistent with what is observed in patients with ASD, including aberrant social interactions, increased repetitive behaviors, and changes in vocalizations (Tsai et al., 2012). Subsequent studies showed that genetically altering TSC1 *in vivo* also induced structural connectivity defects within cortical areas that have been suspected to be dysfunctional in patients with ASD (Stoodley et al., 2018). These studies were among the first to mechanistically link Purkinje cell dysfunction with specific neurobehavioral outcomes in ASD and support the interesting hypothesis that dysfunction in cerebellar computations, that are perhaps localized to specific lobules such as right CrusI/II, could lead to motor as well as cognitive dysfunction.

The social motivation hypothesis in ASD extends on the findings of altered affective behavior in these patients by positing that the aberrant social interaction is due to inability to appropriately assign reward to social interactions (Dawson et al., 2004; Dawson and Webb, 2010). In fact, Purkinje cell function has been directly linked to prefrontal cortical dopamine release, which could be rescued with direct stimulation of the cerebellar nuclei, bypassing Purkinje cell output (Mittleman et al., 2008). Recently, this cerebellar control over the reward circuitry has been tied to direct connectivity between the cerebellar nuclei and the ventral tegmental area (VTA), a canonical reward center, in the mouse (Carta et al., 2019). Strikingly, modulation of cerebellar connectivity to the VTA was sufficient to alter social behavior in the mouse (Carta et al., 2019). This work points to a surprising level of non-canonical network connectivity that may provide key insight into the role of the cerebellum in ASD and other neuropsychiatric disorders in which abnormal reward response to social interactions is thought to play a role in disease manifestation.

## Interregional Connectivity and the Cerebellum

The preceding discussion, which emphasizes that contribution of the cerebellum to cognitive processes, may give the unwanted impression that the cerebellum operates as a regulator of cognitive processes to the exclusion of motor function. This, however, is likely not the case. In fact, the integral role of the cerebellum to motor function is canon (Manto et al., 2012; Lang et al., 2017). Rather, the implication of the above discussion is that the cerebellum may actually serve to regulate distributed cortical function via extensive interregional connectivity to nearly all areas of the neocortex (Caligiore et al., 2017; Bostan and Strick, 2018; Miterko et al., 2018; Diedrichsen et al., 2019), whereby diseases with primarily, though not exclusive, cognitive morbidity such as ASD and PFS may have significant cerebellar involvement. With this in mind, the concluding portions of the review will cover evidence supporting a central role for the cerebellum in distributed cortical function, including anatomical, functional, and evolutionary perspectives.

Over 125 years ago, one of the early debates in neuroscience included the discussion of localizationism championed by Charcot in opposition to a proto-network formulation positing distant effects to local lesions championed by Brown-Sequard (Carrera and Tononi, 2014). The latter theory was reformulated as *diaschisis* by von Monakow, a concept that included the idea that focal lesions led to distant physiologic and clinical effects that could evolve over time (Carrera and Tononi, 2014). This concept was difficult to validate until the advent of more advanced imaging that could assay brain metabolism after brain lesions (Raichle et al., 1975; Phelps et al., 1979). In fact, one of the earliest radiographic examples of diaschisis was found in the cerebellum with an entity described as crossed cerebellar diaschisis, in which hypometabolism is observed in cerebellar hemispheres contralateral to a supratentorial lesion (Baron et al., 1981). Since that time, clinical correlation to this phenomenon has been made including a case report in which there was persistent alterations in cerebellar activity after contralateral basal ganglia infarct (Di Piero et al., 1990), a case series showing that in 20% of patients with thalamic infarct there was associated hypoperfusion of the contralateral cerebellum (Förster et al., 2014), and finally an intriguing case report in which a childhood lesion of the cerebellum corresponded to alterations in contralateral cortical hemispheric function that was compensated by the ipsilateral cortex during performance of a motor task (Nakahachi et al., 2015). The concept of diaschisis is inextricably linked to the concept of distributed network formation, or inter-regional connectivity.

Original conceptualizations of cerebro-cerebellar connectivity regarding cerebellar functional output to the cerebrum were viewed as being restricted to the motor cortex (Allen and Tsukahara, 1974). Over the years, however, our understanding of the connectivity between the cerebellum and cortical structures has expanded immensely to include extensive mono-, di-, and multi-synaptic projections to and from nearly the entire neocortex, thalamus, and basal ganglia (Schmahmann and Pandya, 1993; Caligiore et al., 2017; Shinoda et al., 2017; Kelly and Strick, 2018). Functionally, this organizational structure has expanded to include direct functional correlations between the cerebellum and cerebral cortical structures. While basal ganglia-cortical loops and cerebello-cortical loops had previously been thought to be distinct, evidence emerged for tight anatomical and functional connectivity between the cerebellum and basal ganglia (Chen et al., 2014). Furthermore it has been shown that Purkinje cell activity can reflect oscillatory activity of pre-frontal cortical neurons (McAfee et al., 2019) and that cortical motor learning tightly correlates to granule cell activity (Wagner et al., 2019). The latter two studies reflect intimate bidirectional correlations between the cerebellum and cortex, such that the primary cells receiving cerebral afferents, the granule cells, and the primary modulators of cerebellar efferent signaling, the Purkinje cells, are both implicated in supporting cerebral cortical activity. In fact, almost the entire neocortex has now been mapped into functional domains across the cerebellar cortex (Brissenden et al., 2016; King et al., 2019).

In the setting of human disease, two canonical movement disorders, Parkinson’s disease (PD) and essential tremor (ET), serve as a clinical counterpart to the changing understanding of the role of the cerebellum in distributed cortical network function. While previously thought of as a motor systems disease, PD has emerged as a syndrome encompassing a wide variety of etiologies with symptoms across behavioral domains (Jankovic, 2008; Michel et al., 2016). Similarly, ET, once defined as a benign tremor disorder, has increasingly been associated with non-motor symptoms (Bologna et al., 2019) and may herald more pervasive neurodegenerative disease (Laroia and Louis, 2011; Tarakad and Jankovic, 2018). In particular, patients suffering from either disease, PD or ET, are predisposed to depressive mood changes, sleep disruptions, and cognitive impairments (Puertas-Martín et al., 2016). These symptoms are generally associated with disturbed integrity of prefrontal networks (Puertas-Martín et al., 2016). Interestingly, in these diseases pathologic changes can be found in the cerebellum (Quattrone et al., 2008; Wu and Hallett, 2013; Dyke et al., 2017; Piccinin et al., 2017). Though the precise etiologies of both diseases remain cryptic, the described cerebellar involvement only furthers the notion of intimate connections across broad cortical domains. Moving forward, studies involving functional connectivity in patients suffering from various forms of each disease may help to better understand the contribution of the cerebellum to prefrontal network integrity and function.

That the cerebellum is involved in cortical networks is well grounded in evidence, as described above. However, there is an argument to be made that the cerebellum is actually a crucial node in these networks and is necessary for their structural and functional integrity; this is a claim that is central to the concept of diaschisis. Some evidence to support this claim is as follows: As described in brief on the topic of PFS, requirement of ongoing cerebellar neurotransmission to the survival of olivary neurons has been demonstrated (Ogawa et al., 2010; Patay et al., 2014; Konno et al., 2016; Sabat et al., 2016; Wang et al., 2019). In this case, the disruption of a node (dentate) in the tripartite dentato-rubro-olivary network leads to the transsynaptic degeneration of a second node (olivary) (Sabat et al., 2016). Other examples of degeneration over longer anatomical distances exist, however. In the case of epilepsy, which is thought to be driven primarily by neocortical structures, cerebellar atrophy is a common finding (Hermann et al., 2005; Kros et al., 2015; Allen et al., 2019). This leads to speculation as to whether cerebellar volume changes in certain etiologies of ASD reflect network disruption in addition to those with primary cerebellar pathogenesis (see above discussion).

Finally, recent clinical and model organism studies have begun to demonstrate efficacy of cerebellar stimulation in motor recovery after ischemic stroke, leveraging the newfound understanding of inter-regional connectivity and its role in supporting the integrity of cortical networks (Wessel and Hummel, 2018; Miterko et al., 2019). In rodents, several studies have demonstrated improved motor recovery using cerebellar stimulation after induced cerebral ischemia (Cooperrider et al., 2014; Shah et al., 2017; Chan et al., 2018). Mechanistically, this cerebellar stimulation has been linked to perilesional plasticity, neurogenesis, and neuroprotection via upregulation of telomerase at the site of ischemia (Cooperrider et al., 2014; Chan et al., 2018; Zhang et al., 2019). How does the extensive interregional connectivity of the cerebellum suggested by diaschisis and neurologic disease relate to the role of the cerebellum in complex behavior including cognition?

A central tenet of neuroscience has long placed the expansion of the primate neocortex as the cornerstone for the evolution of intelligence. However, as reviewed, experimental and observational studies define a crucial role for the cerebellum in neocortical network function and even the maintenance of cortical network integrity. In fact, comparative evolutionary studies complete this perspective and position the cerebellum as a key brain region that enables behavioral specialization and drives cortical network complexity across vertebrate lineages. The following section will review the arguments that cerebellum may be a permissive component for the evolution of behavioral complexity in vertebrates.

## An Evolutionary Perspective of Gross Cerebellar Architecture: Implications on Behavior

In the first half of the twentieth century, Olaf Larsell characterized and developed the nomenclature of the cerebellar fissures and lobules, recognizing the gross conservation of the structure of the cerebellum from birds through mammals (Larsell, 1937, 1948, 1952). Subsequent work has identified the presence of the cerebellum as a discrete brain structure in all jawed vertebrates, or gnathostomes (Nieuwenhuys et al., 1998; Butts et al., 2011), based on conserved cellular patterning and shared developmental origins (Chaplin et al., 2010). This finding marks the cerebellum as a prominent feature of vertebrate nervous system evolution, as jawless vertebrates (agnatha) comprise only a small portion of all vertebrate species. As described below, the cerebellum displays interesting characteristics that position it as a key player in the diversification of behavior in vertebrates (Montgomery et al., 2012).

### A Digression on the Evolutionary Origins of the Cerebellum

**FIGURE 4:**
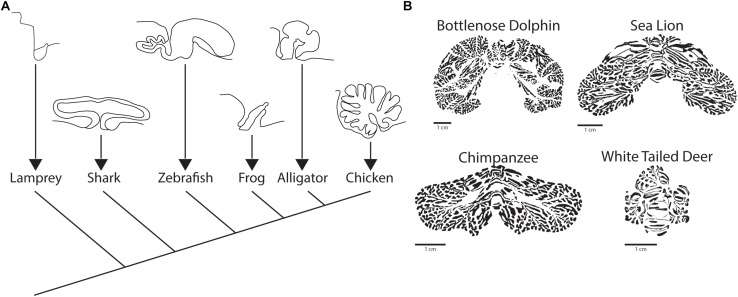
Comparative evolution of the cerebellum. **(A)** Sagittal view of cerebellum and cerebellum-like structures across vertebrates, including a sagittal view of the octavolateralis of the lamprey. Adapted from Butts et al. (2011) with permission obtained from SpringerLink. **(B)** Examples of the posterior cerebella of three species (Bottlenose Dolphin, Sea Lion, Chimpanzee) that display vocal production learning and a single mammal (White Tailed Deer) that does not. Figure inspired by Smaers et al. (2018) but independently processed directly from Brainmuseum.org (Welker et al., 2019) use was as per open source published guidelines for both eLife and Brainmuseum.org, respectively.

Among species within the infraphylum gnathostoma, there is a wide range of cerebellar morphologic variation, ranging from the flat sheet found in amphibians to the ornate, foliated cerebellum of birds and mammals (Figure 4A; Butts et al., 2011). In clades more closely related to mammals, certain aspects of the gross structure of cerebellum are conserved including a midline vermis and rostrocaudally oriented transverse zones. In mammals and birds there are generally four conserved transverse zones, which are described as anterior (AZ), central (CZ), posterior (PZ), and nodular (NZ) (Ozol et al., 1999; Armstrong and Hawkes, 2000). In birds, there is an additional zone referred to as the lingular zone (LZ) (Marzban et al., 2010). The identity of zones is based heavily on the molecular patterning of markers such as Zebrin II.

As mentioned, over the course of vertebrate evolution, the appearance of the cerebellum coincides with the development of the jaw (Striedter, 2005; Montgomery et al., 2012). As would be expected, the cerebellum did not appear *de novo*, but rather seems to have an antecedent in the cerebellum-like octavolateralis nuclei of the agnatha, which are present in the jawless, cerebellum-lacking lamprey as well as the in the jawed, cerebellum-containing chondrichthyans (cartilaginous fishes) (Bell, 2002; Montgomery et al., 2012). The octavolaterlis nuclei have a grossly conserved molecular layer in common with the cerebellum, while lacking Purkinje cells and climbing fiber input; functionally, they seem to operate as purely sensory organs, with particular importance in the mechano- and electro-sensory systems (Yopak and Montgomery, 2008; Montgomery et al., 2012). Once the cerebellum evolved in the jawed vertebrates, its expansion has been correlated to the convergent evolution of complex behaviors across vertebrate phylogeny, as will be detailed below.

Despite the early divergence of the cartilaginous fishes in the vertebrate lineage, the principle that the cerebellum and the telencephalon scale in tandem with increases in brain sizes holds for this clade (Yopak and Montgomery, 2008; Yopak et al., 2010). While more in depth studies, which are technically impractical in these animals, would be required to make direct correlations to specific behaviors in the cartilaginous fishes, there does seem to be a correlation between relative cerebellar size and foliation with environmental enrichment (increased in reef dwelling as compared to deep water species) (Yopak et al., 2010). An interesting speculation that arises from this apparent consistency in brain scaling is that the presence of the cerebellum has been a permissive step for the evolution of complex behavior in vertebrates, and that the correlated relative expansion of the telencephalon with the cerebellum is the material evidence of this cooperation.

The idea that the cerebellum is tightly involved with behavioral specializations in individual species has been more closely studied in animals in which ethologically relevant behavior is tractable to scientific analyses. In particular, both birds and mammals have been evaluated in depth in this regard. Particular species of birds are unique among non-mammalian species as having been found to have a wide variety of apparent higher cognitive function, including tool manufacture, object permanence, and theory of mind (Emery and Clayton, 2004; Güntürkün and Bugnyar, 2016). As described previously in the discussion regarding chondrichthyan brain evolution, a relative expansion of the telencephalon and cerebellum also accompanies increasing behavioral complexity in birds (Gutiérrez-Ibáñez et al., 2018). Furthermore, in bird species with higher cognitive function (crows and parrots) there is a greater cerebellar surface area, which takes into account the folding of the cerebellum (Sultan and Glickstein, 2007).

An interesting contrast between birds and mammals is that while they both have an allometric expansion of the telencephalon and cerebellum, it is only the cerebellum that maintains homologous micro and macrostructure (Gutiérrez-Ibáñez et al., 2018). In fact, birds lack key aspects of the cerebral cortex, which is seen by many as the hallmark of primate cognition (Güntürkün and Bugnyar, 2016). Interestingly, the bird telencephalon connects to the cerebellum via a unique midbrain nucleus, the medial spiriform nucleus, rather than the pontine nuclei as seen in mammals (Gutiérrez-Ibáñez et al., 2018). This observation provides surprising context to the discussion in that the evolution of higher cognition in bird species is convergent on the level of broad network formation, yet nonetheless has adapted the cerebellum as a crucial component of information processing through analogous rather than homologous midbrain nuclei.

### The Mammalian Cerebellum and Expansion of the Neocortex

The hallmark of the mammalian cerebellum is the development of cerebellar hemispheres and the associated development of 10 anterior to posteriorly oriented lobules (Larsell, 1967). Despite almost universally comprising less than 20% of the total brain mass (∼10% in humans) the cerebellum houses well over half of the total neurons (∼80% in humans) in the mammalian brain (Herculano-Houzel et al., 2015). Furthermore, the cerebellum has expanded nearly in proportion to neocortical expansion in higher primates (Smaers, 2014). Even in non-primate mammals, the cerebellar hemispheres demonstrate greater relative expansion in those species that display vocal production learning (Smaers et al., 2018), an assay that serves as a proxy to gauge higher order learning in non-primate species (Janik and Slater, 2000). It is interesting to note that the regional hemispheric expansion is not uniform across species, but rather conforms to the nature of the specialization that the animal uses in its behavior.

One of the more in depth studies on cerebellar specializations in mammals, murine and primate species excluded, comes from the thorough molecular patterning analysis of the star nosed mole by Marzban et al. (2015). The authors undertook this analysis to test the hypothesis that the cerebellar machinery adapts to accommodate specializations that define an animal’s way of life. Consistent with this hypothesis, the cerebellar lobules receiving visual input (those in the central zone) are diminished while those receiving inputs from the trigeminal nucleus (PZ CrusI/II), which is the nucleus that receives mechanosensory information from the “star”, are expanded. Similar domain specific hemispheric expansion is seen in the mammals that have been characterized as having vocal production learning (VPL) (Figure 4B): primates, pinnipeds (fin footed mammals e.g., seals), toothed whales, and elephants (Smaers et al., 2018), although in the latter three more detailed analysis is needed. A final evolutionary observation of the mammalian cerebellum in regards to behavioral specialization that ought to be noted is the presence of a lingular-like zone in lobule I of bats, or microchiroptera (Kim J.Y. et al., 2009; Marzban et al., 2015), which was previously noted not in mammalian but avian cerebellum. It is interesting to speculate on whether this is indicative of another example of convergent evolution, in this case regarding flight, for which the cerebellum is part of the neural substrate.

When considering cognition, higher primates occupy a unique position in that we have insight based on self-reflection while also being able to pull from human disease and primate models to better understand the neural basis of cognition. There is essentially universal agreement that the neocortex plays a central role in the development of higher cognition in humans and higher primates (Rakic, 2009). More recently, evidence from evolutionary analyses have implicated cerebellar expansion as a correlated phenomenon that parallels the expansion of the neocortex in both higher primates, generally, but especially so in humans (Barton and Venditti, 2014). Interestingly, the expansion of the cerebellum in humans correlates with an increase in the number of cerebellar neurons (Herculano-Houzel et al., 2015), while the expansion of the neocortex is correlated with an increase in cortical white matter (Zhang and Sejnowski, 2002; Wang et al., 2008). These correlative studies do not independently comment on function, but perhaps do suggest that increased cortical connectivity and cerebellar information processing are critical components of the higher primate nervous system. Theories on the selective pressures that have driven the evolution of the higher primate CNS are by nature speculative but almost universally implicate the necessity of motor planning requiring the integration visuospatial and motor commands, as is required in ambulation and brachiation, as a pretext to the subsequent development of tool use, extractive foraging, and syntactical language development (Ackermann, 2008; Byrne and Bates, 2010; Londei et al., 2010; Fitch, 2011). This interpretation would necessitate that the cerebellar nuclei, which link the cerebellar cortex to the telencephalon, underwent elaboration in tandem with the expansion of the neocortex and cerebellum itself. Indeed, this seems to be the case. There has been a specific trend toward increasing size of the lateral cerebellar nuclei in primates with a specific and marked increase in the dentate, especially in the ventral portion (Matano and Hirasaki, 1997; Matano, 2001). Detailed analysis of the expansion of the dentate revealed that the increased volume in fact reflected an increase in the surface area of the dentate gray matter (Sultan et al., 2010). Furthermore, there are differences in the cytology of the nuclei when comparing the dorsal to the ventral dentate (Tellmann et al., 2015), the latter of which has projections to associative areas including the prefrontal and posterior parietal cortices via the thalamus (Dum and Strick, 2006). This finding was recently confirmed in humans using associative probability mapping and correlations with volumetric analyses of post-mortem brain tissue (Tellmann et al., 2015).

Despite the canonical belief that the cerebellum was a brain region devoted primarily to motor learning, coordination, and execution, the above described body of literature reflects the more recent understanding that the cerebellum is a critical component of coordinated and integrated cortical function across domains. Furthermore, cerebellar expansion accompanies increasingly complex behavior across jawed vertebrates and may in fact be thought of as a necessary component of higher cognitive function, at least as defined by one cognitive parameter (VPL). Open questions remain regarding the nature of the computations performed, how universal these functions are across domains, and how they specifically work to form what we consider to be normal cognitive function (Diedrichsen et al., 2019). Moreover, an increasing body of literature points to the cerebellum as a key node in the formation and function of cortical networks (Caligiore et al., 2017; Bostan and Strick, 2018; Miterko et al., 2018).

## Conclusion

This review has sought to provide the basis for the emerging consensus that the cerebellum is not only a passive component of cognitive and affective behavior, but a key neural substrate that is actively involved in a great number of fundamental behaviors. The repeated adaptation of the computational circuitry and the molecular diversity of the cerebellum in the evolution of complex behavior among vertebrate species offers a compelling argument that the cerebellum is a key component of behavioral specialization. For humans, the fluid continuity between the rich cognitive subtext of our motivations and desires and the remarkable complexity of our motor output cannot be easily disentangled. In the cerebellum, we find a critical neural substrate that has the capacity to seamlessly coordinate these motor and non-motor functions. It is a crucial time in neuroscientific inquiry to expand upon and utilize our knowledge of this structure to better understand how brain networks are formed and how they function in health and disease. This may help to devise interventions to structurally rewire and correct the disruptions that underlie a wide variety of neurologic and neuropsychiatric diseases.

## Author Contributions

JG and RS wrote and edited the manuscript.

## Conflict of Interest

The authors declare that the research was conducted in the absence of any commercial or financial relationships that could be construed as a potential conflict of interest.
